# *Cephalenchus driekieae* n. sp. (Nematoda: Tylenchidae) from South Africa, a new member of the genus with a long pharyngeal overlap

**DOI:** 10.21307/jofnem-2020-031

**Published:** 2020-04-24

**Authors:** Milad Rashidifard, Gerhard Du Preez, Joaquín Abolafia, Majid Pedram

**Affiliations:** 1 Unit for Environmental Sciences and Management, North-West University, Private Bag X6001, Potchefstroom, 2520, South Africa; 2 Departamento de Biología Animal, Biología Vegetal y Ecología, Universidad de Jaén, Campus Las Lagunillas, s/n; 23071, Jaén, Spain; 3 Department of Plant Pathology, Faculty of Agriculture, Tarbiat Modares University, Tehran, Iran

**Keywords:** Morphology, Molecular identification, Phylogeny, Pleurotylenchinae, Ribosomal DNA, Tylodoridae, Tylodorinae

## Abstract

*Cephalenchus driekieae* n. sp. is described and illustrated based on its morphological, morphometric, and molecular characteristics. This new species is mainly characterized by its short stylet 11.5 to 13.0 μm, and 13.5 to 17.5 μm long pharyngeal overlap extending over the intestine. It could further be delimited by 451 to 526 μm long females with a prominently annulated cuticle, dorso-ventral amphidial openings as shown using scanning electron microscopy (SEM), four lines in the lateral field, anchor-shaped stylet knobs, empty spermatheca, elongate conoid tail with finely rounded tip and males absent. The shortest stylet and long pharyngeal overlap, distinguish this new species from previously described members and update the characteristics of the genus. With four lines in the lateral field, this new species was morphologically compared with four previously described species with this feature and another species with a short stylet. Molecular phylogenetic analyses using the partial small and large subunit ribosomal DNA gene (SSU and LSU rDNA D2-D3) sequences showed that it was clustered with other *Cephalenchus* spp. in both SSU and LSU trees, retaining the monophyly of the genus. This new species from South Africa updates the biogeography of the genus.

The family Tylenchidae (Örley, 1880) is an abundant group of nematodes. Its members are commonly found in vast ecological niches *viz*. soil, algae, fungi, and plant material (Panahandeh et al., 2018). Currently, approximately 44 genera and 412 species are known in this family (Qing and Bert, 2019). Traditionally, most tylenchids were identified based solely on morphological characters (Geraert, 2008), which led to taxonomic confusions within the family (Qing and Bert, 2019). However, in recent years, DNA-based techniques have been extensively used for taxonomic studies of tylenchids (Bert et al., 2010; Pereira and Baldwin, 2016; Pereira et al., 2017; Pedram et al., 2018; Panahandeh et al., 2018, 2019; Qing and Bert, 2019).

*Cephalenchus* (Goodey, 1962) was proposed as a subgenus of *Tylenchus* (Bastian, 1865; Goodey, 1962). Later, Golden (1971) elevated it to the genus level with *Cephalenchus hexalineatus* (Geraert, 1962; Geraert and Goodey, 1964) as its type species. Currently, 20 species have been described under the genus *Cephalenchus* most of which were established based on traditional criteria (Geraert, 2008; Pereira et al., 2017). Although some species have been reported from Australia, New Zealand, Chile, and Congo, the genus mostly occurs in the northern hemisphere (Geraert, 2008; Pereira et al., 2017). This is at first due to sampling bias, which requires further sampling efforts to achieve a potential wider distribution map of the genus across the world. In recent years, two phylogenetic studies have focused specifically on *Cephalenchus*, investigating several aspects of its evolution including inter- and intraspecies and intragenomic variations, correlation of morphology and phylogeny and biogeography (Pereira and Baldwin, 2016; Pereira et al., 2017).

During our recent surveys in South Africa, a population of *Cephalenchus* with a remarkably short stylet, long pharyngeal overlap, and four lines in the lateral field was recovered from the rhizosphere of grasses near a maize field, 20 km north of the town Lichtenburg (North-West Province, South Africa). In-depth investigations revealed that it belongs to an unknown species, being described herein as *C. driekieae* n. sp. Therefore, the present study aims to characterize this species using both traditional and molecular techniques and discuss its phylogenetic affinities using SSU and LSU markers.

## Materials and methods

### Nematode extraction and morphological studies

Nematodes were extracted from the soil using the modified sugar centrifugal flotation method (Jenkins, 1964). The specimens were fixed in hot formalin and transferred to anhydrous glycerin for slide preparation following De Grisse (1969). Measurements and drawings of the mounted specimens were done using a Nikon Eclipse E600 light microscope. Photographs were taken using an Olympus DP72 digital camera attached to an Olympus BX51 microscope equipped with differential interference contrast (DIC) optics. Digital drawings were made using the hand-made drawings as template to redraw in CorelDRAW^®^ software version 2018.

#### Scanning electron microscopy (SEM)

For SEM, the nematodes mounted on permanent slides were cleaned and hydrated in distilled water, dehydrated in a graded ethanol-acetone series, critical point dried, coated with gold, and observed with a Zeiss Merlin microscope (5 kV) (Zeiss, Oberkochen, Germany) (Abolafia, 2015).

### DNA extraction, PCR reaction, and gel electrophoresis

DNA extraction and PCR conditions followed Rashidifard et al. (2019). The following primers were used for amplification and sequencing: SSU F04 (GCTTGTCTCAAAGATTAAGCC) and SSU R26 (CATTCTTGGCAAATGCTTTCG) (Blaxter et al., 1998) for SSU rDNA; and D2A (5´-ACAAGTACCGTGAGGGAAAGTTG-3´) and D3B (5´-TCGGAAGGAACCAGCTACTA-3´) (Subbotin et al., 2006) for D2-D3 LSU rDNA.

Four microliters of PCR products were loaded on a 1% agarose gel (40 mMTris, 40 mM boric acid, and 1 mM EDTA) to check the quality of the amplified DNA. The DNA bands were stained with GelRed and visualized and photographed using a UV transilluminator. The PCR products were sequenced in both directions by the genomic company Inqaba biotec™ (South Africa; www.inqaba-southafrica.co.za).

### Phylogenetic analyses

The newly obtained sequences were compared with those of other species available in GenBank using the BLAST homology search program. For reconstructing the SSU and LSU trees, several sequences were retrieved from the database and the newly generated sequences were included. The SSU data set was aligned using MUSCLE (Edgar, 2004) as implemented in MEGA6 (Tamura et al., 2013) and edited manually. The LSU data set was aligned using the G-INS-i algorithm in MAFFT version 7 (http://mafft.cbrc.jp/alignment/server/) (Katoh and Standley, 2013) and post edited using the Gblocks program (version 0.91b) with all the three less stringent parameters (http://phylogeny.lirmm.fr/phylo_cgi/one_task.cgi?task_type=gblocks). The nucleotide substitution model was selected using MrModeltest 2 (Nylander, 2004). The general time reversible model with proportion of invariable sites and a gamma distribution (GTR + I + G) was used in both SSU and D2-D3 LSU analyses. A Bayesian analysis was performed in MrBayes v3.1.2 (Ronquist and Huelsenbeck, 2003) using a random starting tree for each locus and running the chains for 3 × 10^6^ generations for both the SSU and LSU data sets. After discarding burn-in samples, the remaining samples were retained for further analyses. The Markov chain Monte Carlo (MCMC) method within a Bayesian framework was used to estimate the posterior probabilities of the phylogenetic trees (Larget and Simon, 1999) using the 50% majority rule. Convergence of model parameters and topology were assessed based on average standard deviation of split frequencies and potential scale reduction factor values. Adequacy of the posterior sample size was evaluated using autocorrelation statistics as implemented in Tracer v.1.6 (Rambaut and Drummond, 2009). Aphelenchid and neotylenchid outgroup taxa were used for SSU and LSU phylogenies, respectively, based on previous studies (Yaghoubi et al., 2016; Soleymanzadeh et al., 2016; Pereira et al., 2017) (species names and accession numbers listed in corresponding trees). The output ﬁles of the phylogenetic programs used herein were visualized using Dendroscope v.3.2.8 (Huson and Scornavacca, 2012) and re-drawn in CorelDRAW software version 2018.

## Results

Systematics

**Figure 1:**
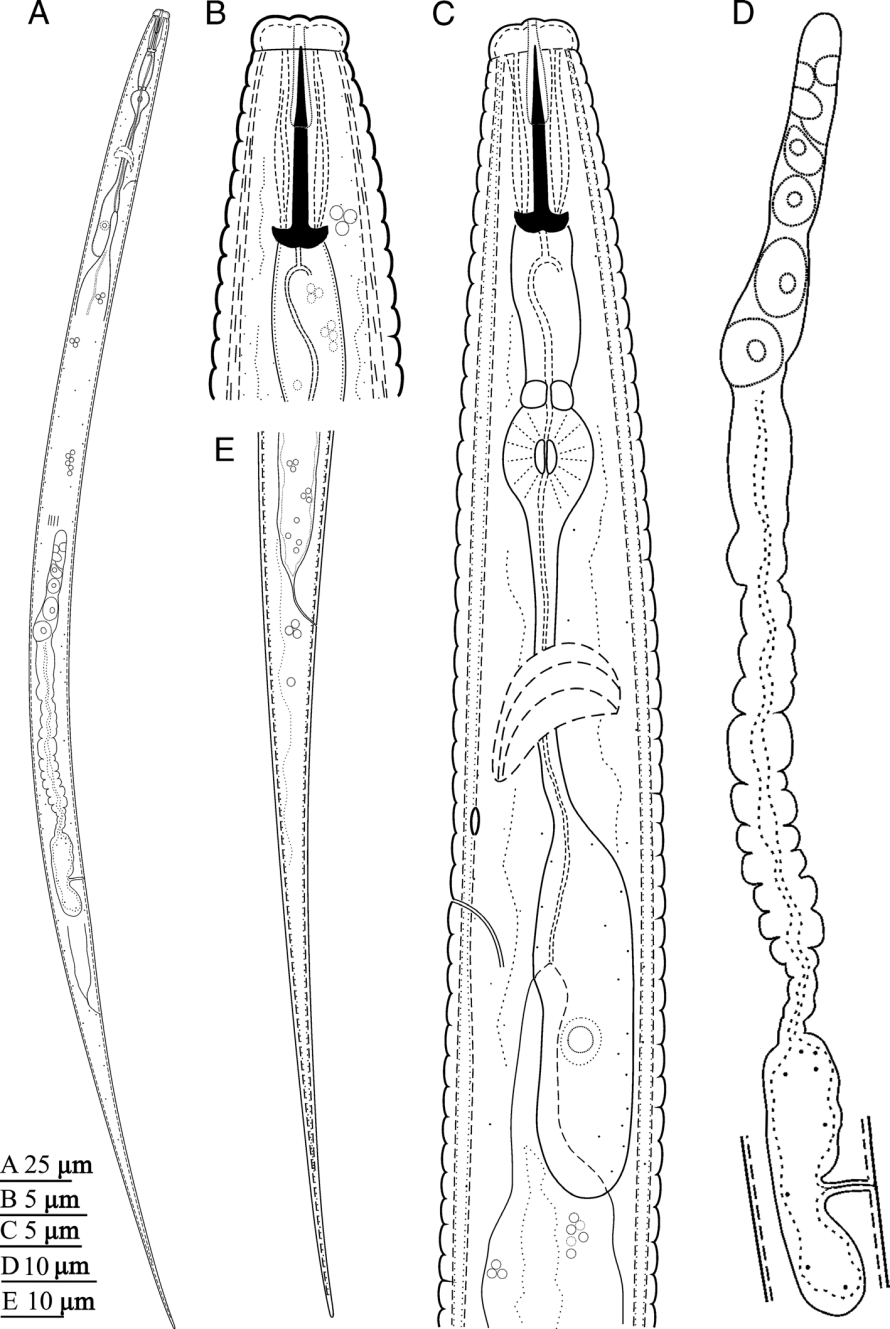
Line drawings of *Cephalenchus driekieae* n. sp. from South Africa, female. (A) Entire body; (B) anterior region; (C) pharyngeal region; (D) reproductive system; (E) tail.

**Figure 2:**
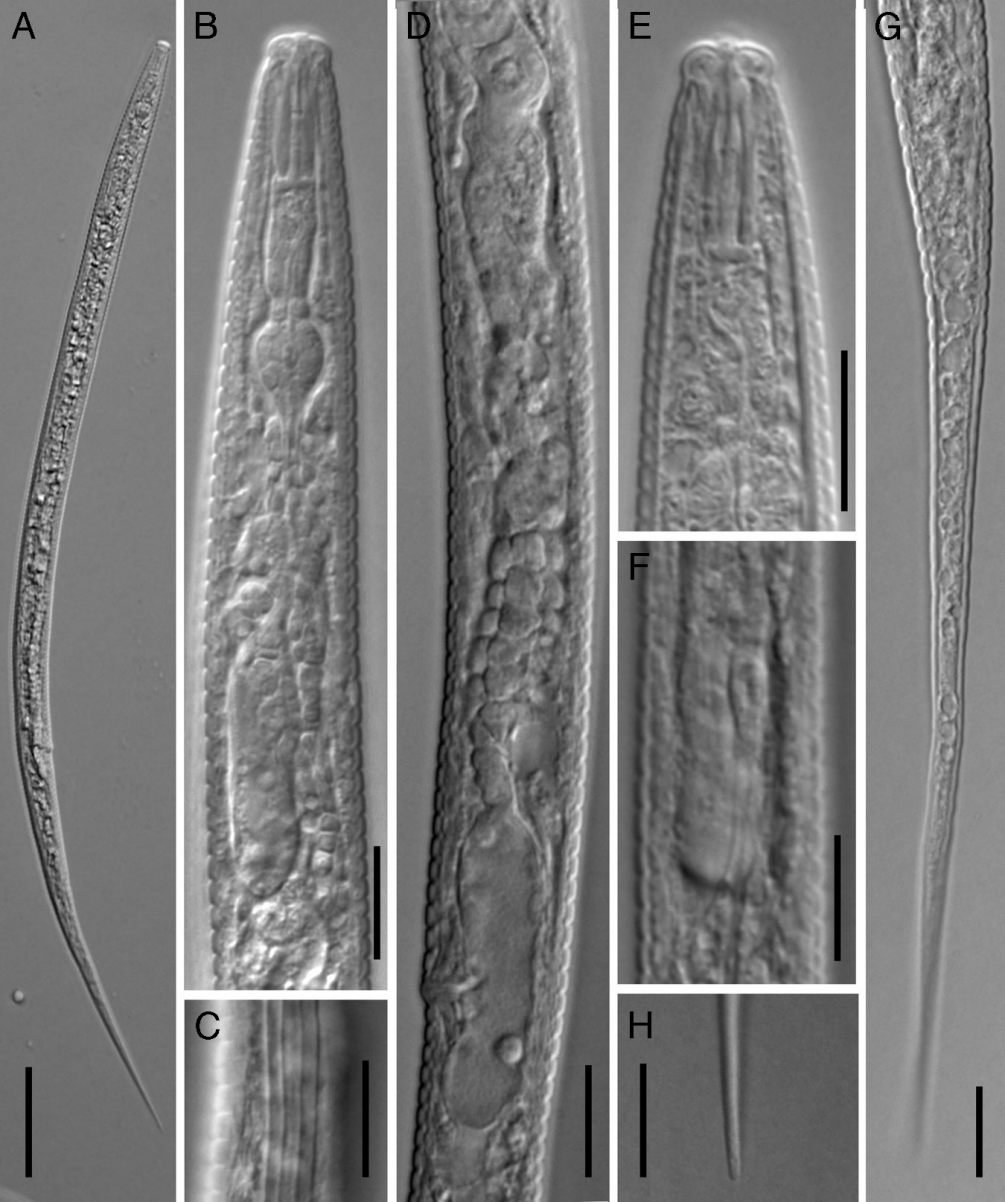
Light micrographs of *Cephalenchus driekieae* n. sp. from South Africa, female. (A) Entire body; (B) pharyngeal region; (C) lateral lines; (D) part of reproduction system; (E) anterior region; (F) pharyngeal overlap; (G) tail; (H) tail terminus. (Scale bars = 10 μm).

**Figure 3:**
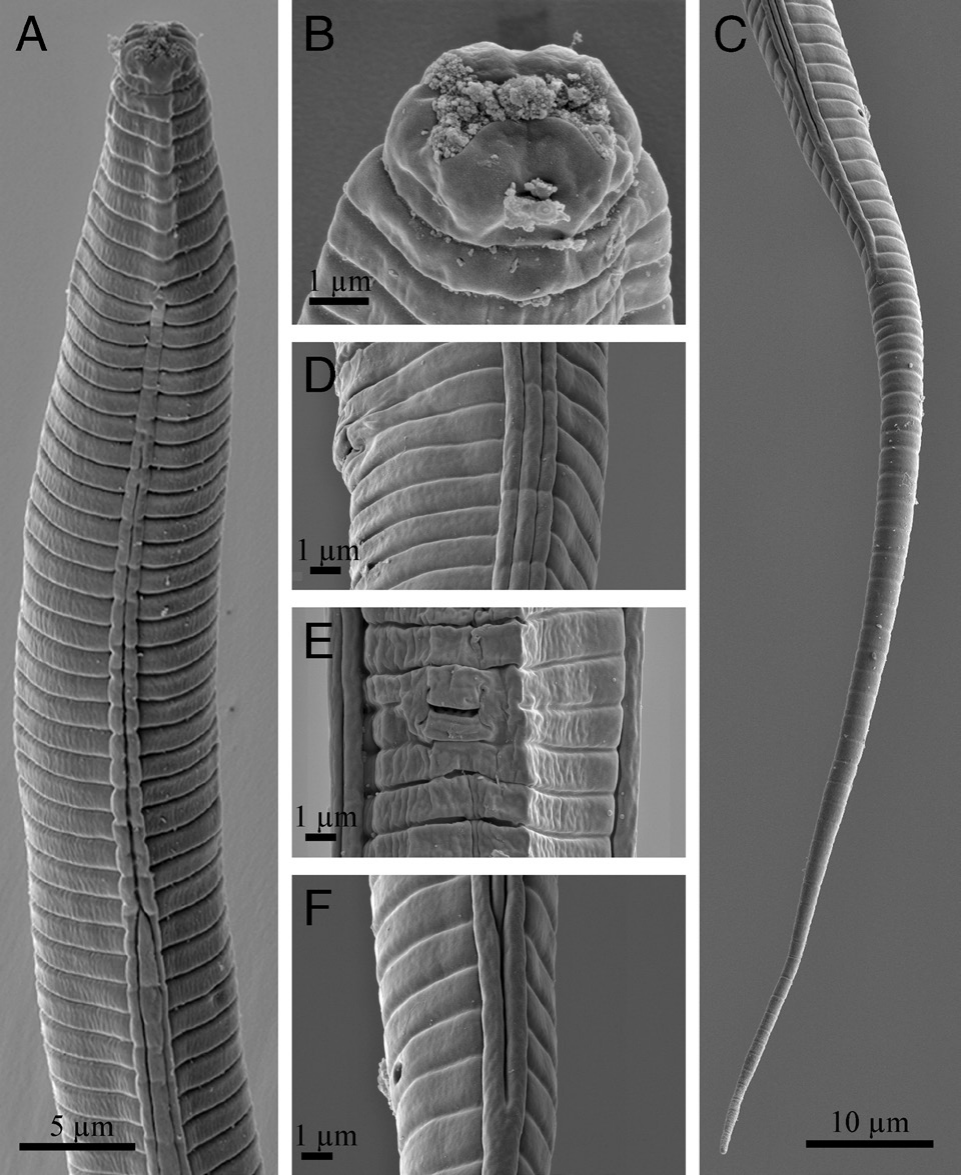
Scanning electron microscopy of *Cephalenchus driekieae* n. sp. from South Africa, female. (A) Anterior region; (B) lip region; (C) posterior body region; (D, E) vulval region in lateral and ventral view; (F) anus and lateral lines in anus region.

Cephalenchus driekieae* n. sp.

(Figs. 1–3; Table 1).

**Table 1.** Morphometric measurements of *Cephalenchus driekieae* n. sp. from South Africa.

	Female	
Characters	Holotype	Paratypes
n	1	8
L	456	483.5 ± 24.5 (451–526)
a	36.5	34.8 ± 1.3 (33.3–36.5)
b	7	7.3 ± 0.5 (6.8–8.0)
c	4	4.3 ± 0.1 (4.1–4.5)
c´	12.2	13.6 ± 1.3 (11.5–15.0)
V	65.8	64.8 ± 2.0 (60.5–67.0)
V´	86.5	83.0 ± 3.3 (75.5–86.5)
Lip region height	2	2.4 ± 0.4 (2–3)
Lip region width	5.5	6.0 ± 0.5 (5.5–7.0)
Stylet conus length	5	5.4 ± 0.6 (4.4–6.3)
Stylet length	12	12.0 ± 0.5 (11.5–13.0)
DGO	1.8	1.3 ± 0.3 (1.0–1.8)
Median bulb valve to ant. end	31	31.0 ± 0.5 (30–32)
MB%	6.8	6.5 ± 0.3 (6–7)
Pharynx (stylet knobs to pharynx-intestinal junction)	56	64.5 ± 5.0 (56–71)
Neck (anterior end to pharynx-intestinal junction)	70	65 ± 3 (61–70)
Excretory pore – ant. end	–	61.5 ± 3.0 (57–65)
Hemizonid – ant. end.	49	58 ± 5 (49–62)
Anterior end to vulva	300	313 ± 22 (280–353)
– to anus	346	372 ± 20 (344–393)
Body width at median bulb	11	12.0 ± 0.5 (11–13)
– at vulva	12.5	14 ± 1 (12.5–15.5)
– at anus	9	8.5 ± 1.0 (7.5–10.0)
Post-vulval uterine sac length	13	13.5 ± 0.5 (13–14)
Tail length	110	111.0 ± 3.5 (105–116)

**Note:** Measurements are in μm and in the form: mean ± standard deviation (range).

Description

### Female

Body slightly ventrally arcuate after fixation, the curvature greater in posterior region, tapering slightly toward posterior end by having an elongate filiform tail. Cuticle thin and coarsely annulated, annuli 1.8 to 2.2 μm wide at mid-body. Lateral fields with three alae or four longitudinal incisures, the outer ones smooth in SEM, ending at proximal part of tail. Cephalic region separated from the rest of the body by a depression, the corners rounded, and its cephalic framework weakly sclerotized. Amphidial openings dorso-ventrally oriented on labial disc (disc, *sensu* Pereira *et al.*, 2016). There are four cephalic papillae on the corners of the labial disc. Oral disc small and rounded. Stylet with a conus ca. 45% of its total length, a cylindrical shaft, and anchor-shaped knobs. The protractor muscles well developed. Dorsal gland orifice located at a short distance to the knobs. Pharynx with wide, muscular and short procorpus, about one stylet length. The constraining muscle at the junction with metacorpus prominent and metacorpus pyriform, 7 to 10 μm long, 5 to 7 μm wide, with a centrally located small valve; isthmus remarkably narrow (especially when compared with procorpus) and slender, pharyngeal glands with long dorsal overlap over the intestine, the overlap 13.5 to 17.5 μm long, the pharyngeal junction with the intestine discernible. Nerve ring at around middle isthmus, 40 to 53 μm from anterior end or ca 70% of the neck length. Excretory pore at anterior half of the pharyngeal glands, or at ca 90% of the neck length. Hemizonid discernible, slightly anterior to the excretory pore. Intestine simple. Rectum about as long as anal body width. Anus distinctive. Reproductive system monodelphic-prodelphic, 117 to 218 μm long (excluding post-vulval uterine sac, PUS); the ovary outstretched, and oocytes behind the germination zone in single row; oviduct tubular and short; spermatheca spherical and empty; uterus with tubular distal part (uterine crustaformeria) and quadricolumellate, with apparently six cells in each row, and proximal part (ovejector) swollen, with thinner walls; PUS spacious, about as long as vulval body width, vagina perpendicular to body axis, 31 to 43% of corresponding body width long and not heavily sclerotized; vulva a small transverse slit and vulval flaps visibly very small under SEM. Tail elongated and filiform, its tip finely rounded.

### Male

Not found.

#### Type habitat and locality

The new species was recovered from soils collected in a small section of a grassland located next to a maize field. This grassland is located 20 km north of the town Lichtenburg (North-West Province, South Africa) at the following GPS coordinate: 26°02´36.31˝S; 26°08´30.57˝E.

#### Type material

Holotype and three paratype females on slide 50945 were deposited in the National Collection of Nematodes (NCN), ARC-PPRI, Pretoria, South Africa. Four paratype females were deposited in the WaNeCo collection, Wageningen, the Netherlands (http://www.waneco.eu/). The LSID for this publication is: urn:lsid:zoobank.org:pub:1F7FFCE8-AE69-4EC0-B6AD-E7A015AF8AD5.

#### Etymology

The species is named after Professor Driekie Fourie who trained several nematologists and dedicated her entire scientific career to the development of nematology and agriculture in Africa.

#### Diagnosis and relationships

*Cephalenchus driekieae* n. sp. is mainly characterized by its pharyngeal overlap of 13.5 to 17.5 μm long and short stylet of 11.5 to 13.0 μm long. It is further characterized by 451 to 526 μm long females having prominently annulated cuticle, four lines in the lateral field, dorso-ventral amphidial openings, stylet with anchor-shaped knobs, pyriform metacorpus, very small vulval flaps in SEM, empty spermatheca, parthenogenetic reproduction mode, 105 to 116 μm long tail with finely rounded tip and a geographical distribution in South Africa. By having four lines in the lateral field, the new species was morphologically compared with four nominal four-lined species *viz*. *C. concavus* (Xie and Feng, 1994), *C. imphalus* (Dhanachand et al., 1993), *C. indicus* (Dhanachand and Jairajpuri, 1980; Raski and Geraert, 1986), and *C. intermedius* (Kanwar et al, 1990), which could be separated from them by a shorter stylet and a long pharyngeal overlap. It was also morphologically compared with *C. tahus* (Wood, 1973) having a short stylet, although it could be separated from this species by a long pharyngeal overlap. The detailed comparisons follow (the data of the compared species after Geraert, 2008).

*Cephalenchus driekieae* n. sp. is different from *C. concavus* by its shorter body (451-526 vs 650-750 μm), stylet (11.5-13.0 vs 22.0-22.5 μm), and tail (105-116 vs 134-190 μm). It can be distinguished from *C. imphalus* by shorter stylet (11.5-13.0 vs 17.0-17.5 μm), very small vulval flap (*vs* large), and shorter tail (105-116 vs 115-155 μm). *C. driekieae* n. sp. differs from *C. indicus* by shorter body (451-526 vs 680-790 μm), very small vulval flap (*vs* large), shorter stylet (11.5-13.0 vs 18-22 μm) and tail (105-116 vs 184-202 μm). Likewise, the new species is different from *C. intermedius* by shorter body (451-526 vs 670-790 μm), very small vulval flap (vs small), shorter stylet (11.5-13.0 vs 16-18 μm), greater V (60.5-67.0 vs 55-58) and shorter tail (105-116 vs 214-280 μm). And finally, it differs from *C. tahus*, by shorter body (451-526 vs 560-660 μm), four lines in lateral field (vs six) and shorter tail (105-116 vs 214-280 μm).

#### Molecular characterization

A BLAST search using the partial SSU rDNA of the new species (899 nt long, MN519728) revealed that it has at maximum 96.33% identity with *Cephalenchus hexalineatus* (KJ869347, KJ869346). A BLAST search using the partial D2-D3 LSU rDNA of the new species (683 nt long, MN519727) revealed it has at maximum 82.29% identity with *C. cephalodiscus* (Sultan and Jairajpuri, 1982) (KU723097).

The SSU alignment of the selected sequences included 1,774 characters of which 607 were variable, and the LSU alignment of the selected sequences included 577 characters of which 411 were variable.

Two SSU rDNA (MN519728 and MN847693) and two LSU rDNA D2-D3 (MN519727 and MN847694) sequences were generated for the new species using four different female specimens. There were no differences between the two SSU and two LSU rDNA sequences. Subsequently, only one sequence from each fragment was used in corresponding phylogenetic trees.

**Figure 4:**
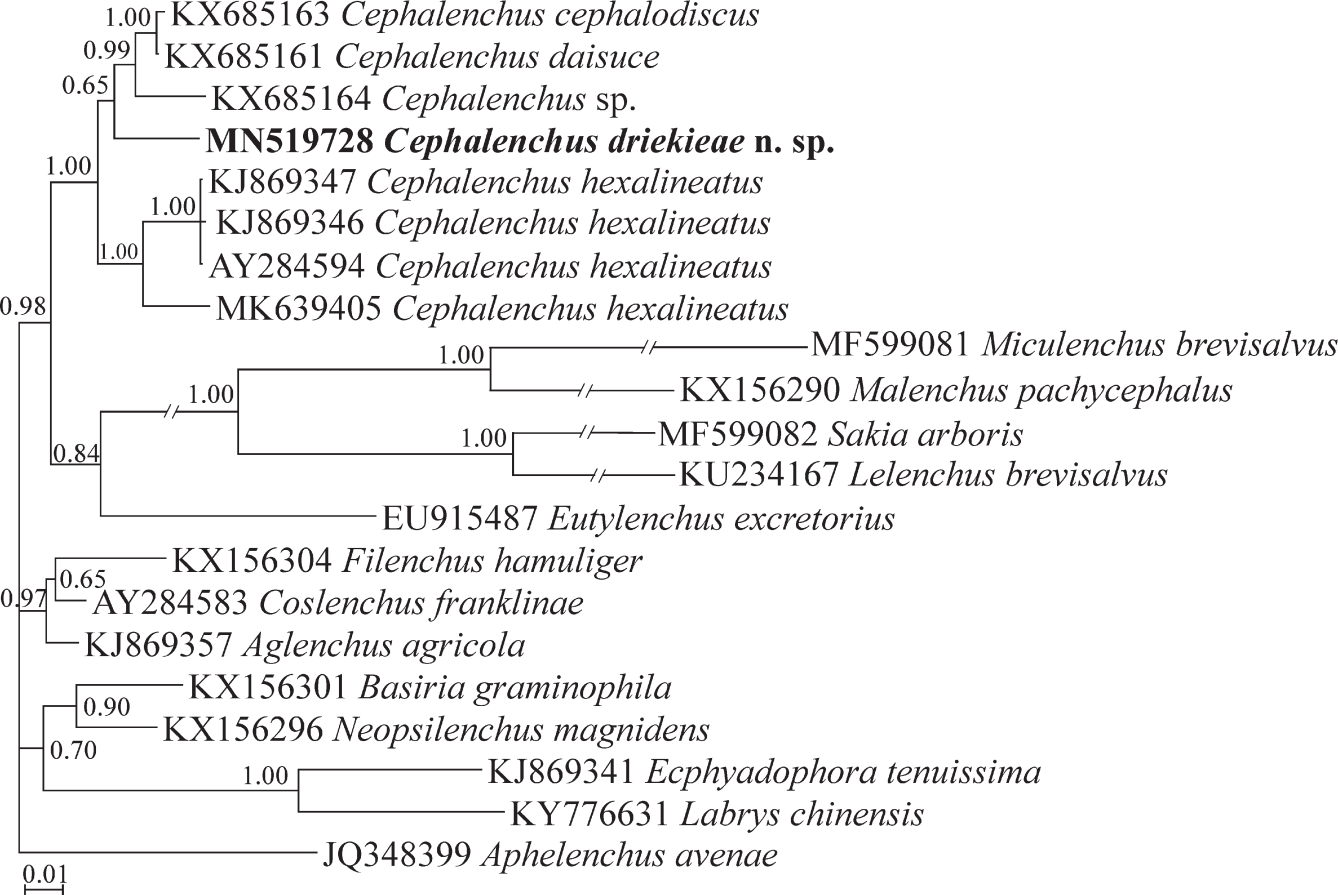
Bayesian 50% majority rule consensus tree inferred from small subunit (SSU) rDNA gene sequence of *Cephalenchus driekieae* n. sp. from South Africa under GTR + I + G model (lnL = 6,436.7202; freqA = 0.2443; freqC = 0.2268; freqG = 0.2864; freqT = 0.2425; rAC = 1.0699; rAG = 2.8891; rAT = 1.2180; rCG = 1.1273; rCT = 5.8691; Pinv = 0.4073; Alpha = 0.6044). Bayesian posterior probability (BPP) values *>*0.50 are given for appropriate clades. The sequence of the new species is indicated by bold font.

In the inferred SSU tree using the above-mentioned SSU data set (Fig. 4), the new species placed into the maximally supported clade of *Cephalenchus*. Within this clade, the new species is in a basal position to three species of the genus.

**Figure 5:**
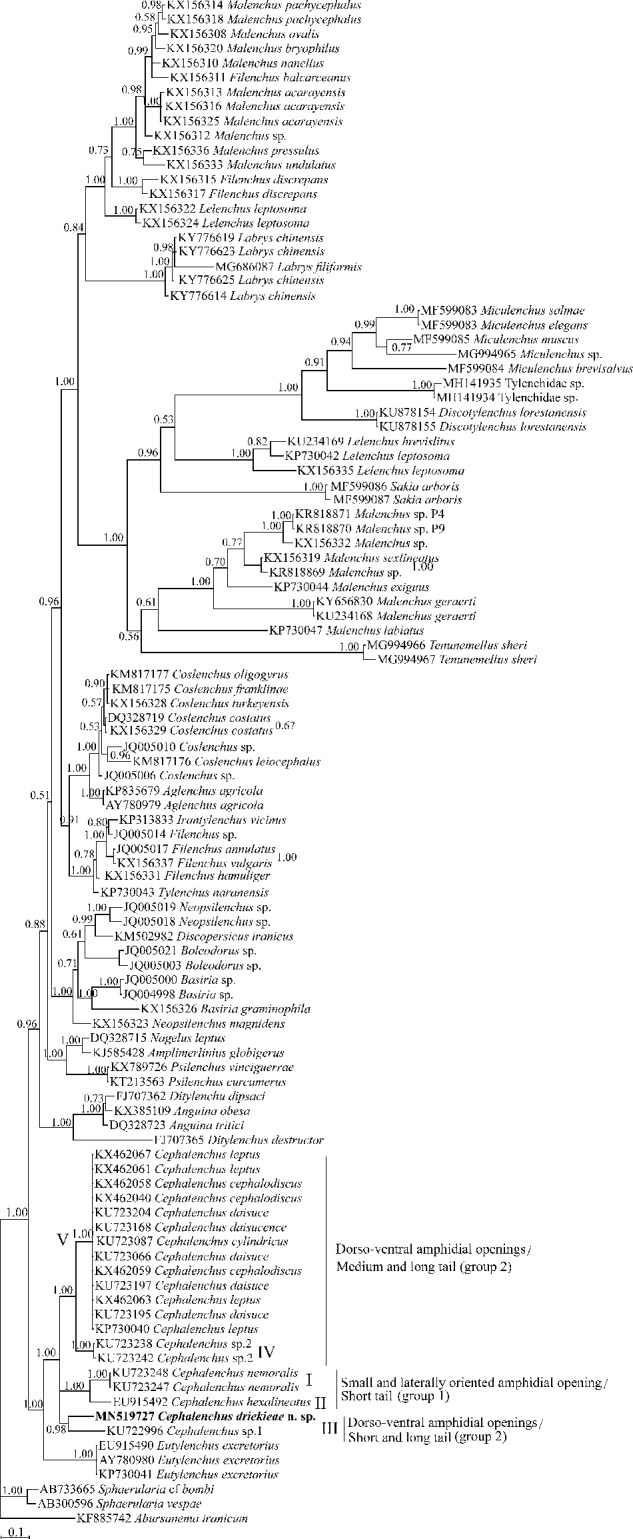
Bayesian 50% majority rule consensus tree inferred from D2-D3 expansion segments of large subunit (LSU) rDNA gene sequence of *Cephalenchus driekieae* n. sp. from South Africa under GTR + I + G model (lnL = 7,632.2964; freqA = 0.1794; freqC = 0.2463; freqG = 0.3573; freqT = 0.2170; rAC = 0.9583; rAG = 2.6626; rAT = 1.2424; rCG = 0.6711; rCT = 5.4981; Pinv = 0.2528; Alpha = 0.6707). Bayesian posterior probability (BPP) values *>*0.50 are given for appropriate clades. The sequence of the new species is indicated by bold font.

In the phylogenetic tree inferred using the above-mentioned LSU data set (Fig. 5), *Cephalenchus* spp. formed a well-supported clade, including the new species. This clade is in sister relation with *Eutylenchus excretorius* (Ebsary and Eveleigh, 1981), both of which occupy a basal placement to other ingroup *Tylenchoidea* spp. The *Cephalenchus* sp. 1 from Brazil (KU722996) (Pereira et al., 2017) is the closest relative of the new species in this tree.

## Discussion

A population of *Cephalenchus*, representing a new species, was recovered in South Africa and described herein as *C. driekieae* n. sp. It has the shortest stylet of the genus and a long pharyngeal overlap, both features delimiting it and updating the characteristics of the genus. As far as we know, this trait (long pharyngeal overlap) is a new characteristic of the family Tylenchidae. The new species has a short tail (105-116 μm), which also occurs in other species of the genus (*C. brevicaudatus* Raski and Geraert, 1986 has a 54-92 μm long tail and *C. hexalineatus* has an 80-140 μm long tail) (Geraert, 2008). Morphological delimitation of *Cephalenchus* spp. mostly relies on lateral line number and tail length, dividing the species into three short, medium, and long tailed forms (Geraert, 1968; Raski and Geraert, 1986; Geraert, 2008). Although clear SEM photos were not successfully prepared for the new species (as the amphidial openings were not clean), the new species has dorso-ventral amphidial openings.

The monophyly of the genus was already shown using different data sets (Pereira et al., 2017). In the present study, the monophyly of the genus was retained after adding the new species in both SSU and LSU phylogenies. The topology of the resolved LSU phylogeny more or less resembles the topology resolved by Pereira et al. (2017).

In the present LSU phylogeny, the new species and *Cephalenchus* sp.2, two clade-III-species members *sensu* (Pereira et al., 2017) having four lines in lateral field have occupied distant placements, showing the lateral line number, in contrast to amphidial opening shape, does not reflect the phylogeny of the genus and could be a convergent trait (Pereira et al., 2017). The two types of amphidial openings of the genus are the lateral small slit, and the large dorso-ventral types. The currently sequenced members of *Cephalenchus* for their LSU fragments are divided into five clades in Figure 5 in accordance with Pereira et al. (2017). *Cephalenchus driekiae* n. sp. belongs to subclade III, currently including the new species and *Cephalenchus* sp. 1, both having the similar amphidial opening, further corroborating the congruence of the amphidial opening shape and the phylogeny.

In previous molecular phylogenetic analyses, different placements for *Cephalenchus*, *Eutylenchus excretorius* or *Cephalenchus* + *Eutylenchus excretorius* clade were resolved using different markers (Subbotin et al., 2006; Bert et al., 2008; Holterman et al., 2009; Palomares-Rius et al., 2009; Panahandeh et al., 2015; Pereira et al., 2017). Moreover, questions already being raised regarding the placement of these two genera in Tylenchoidea (Örley, 1880; Siddiqi, 2000; Subbotin et al., 2006). In conclusion, and in agreement with the aforementioned recent studies, we believe further analyses are required before making a rigid decision on the phylogeny of these two genera.
